# Late Palaeolithic cave art and permafrost in the Southern Ural

**DOI:** 10.1038/s41598-018-30049-w

**Published:** 2018-08-13

**Authors:** Yuri Dublyansky, Gina E. Moseley, Yuri Lyakhnitsky, Hai Cheng, Lawrence R. Edwards, Denis Scholz, Gabriella Koltai, Christoph Spötl

**Affiliations:** 1 Institute of Geology, Innsbruck University, Innsbruck, Austria; 2 A.P. Karpinsky Russian Geological Research Institute, St. Petersburg, Russia; 3 Institute of Global Environmental Change, Xi’an Jiaotong University, Xi’an, China; 4 Department of Earth Sciences, University of Minnesota, Minneapolis, USA; 5 Institute of Geosciences, Gutenberg University, Mainz, Germany

## Abstract

Shulgan-Tash (also known as Kapova) cave located on the western slope of the Ural Mountains (Russia) is the easternmost European cave art monument of late Palaeolithic age. Radiocarbon dates from cultural layers in the cave suggest an age of about 16.3 to 19.6 ka (cal BP), but dates directly on the paintings were not obtained. In order to constrain the age of this art using an independent method, we performed detailed ^230^Th-U dating of calcite flowstone underlying and overgrowing the paintings at 22 sites in three halls of the cave. The youngest age for the underlying calcite (i.e., the maximum age of the cave art) is 36.4 ± 0.1 ka, and the oldest overlying calcite (constraining the minimum age of the cave art) is 14.5 ± 0.04 ka. The ca. 21.9 ka-long hiatus in calcite deposition during which the paintings were made is attributed to regional permafrost conditions and sub-zero temperatures inside the cave during Marine Isotope Stage (MIS) 2. This is supported by samples of cryogenic cave calcite, which document seven episodes of freezing and thawing of permafrost associated with stadials and interstadials of MIS 3, respectively.

## Introduction

From the discovery of parietal art in Altamira cave in northern Spain in 1879 until the mid-20^th^ century, Palaeolithic cave art was thought to be a phenomenon restricted to the Franco-Cantabrian region of Western Europe. In 1959, however, cave art of Palaeolithic age was discovered in Shulgan-Tash (also known as Kapova) cave in Southern Ural, Russia, more than 4000 km further east. This cave, located on the western slope of the Ural Mountains, remains the easternmost occurrence of Palaeolithic cave art that is known about in Europe today.

**Figure 1:**
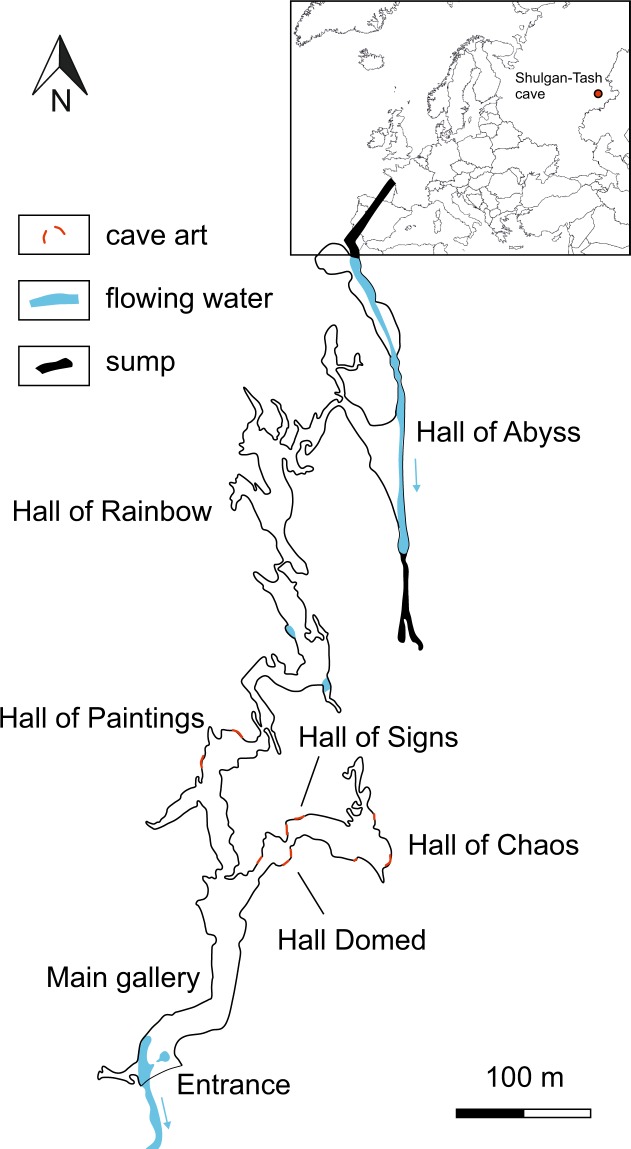
Map of Shulgan-Tash cave. Locations of cave art are shown. Modified from^18^.

Shulgan-Tash cave is located in the Republic of Bashkortostan, Russia, within the Shulgan-Tash Nature Reserve (53.044 N, 57.064 E). The cave is located in the Saryk-Oskan massif, which rises ca. 120 m above the Belaya River. Palaeolithic parietal art is present in four halls of the cave: Domed Hall, Hall of Signs, Hall of Chaos on the lower floor of the cave, and Hall of Paintings on the upper floor (Fig. 1). Palaeolithic paintings were discovered in Shulgan-Tash cave in 1959 by Alexander Riumin, a staff zoologist of the Nature Reserve^1^. The discovery was initially met with scepticism by archaeologists, but esteemed archaeologist Otto Bader, who came to examine the paintings, immediately realised their genuine nature; he spent the subsequent 17 years studying the cave and its paintings^2,3^. Most of the paintings are made using natural red paint (ochre; primary pigment is haematite) sometimes with additions of charcoal^4^. Some 26 figurative paintings depict elements of the “mammoth fauna” (woolly mammoth, horse, woolly rhinoceros, steppe bison, and wisent). In November 2017, after removal of a layer of flowstone overgrowth, a painting of a Bactrian camel was found^5^. Additionally, several anthropomorphic/zoomorphic figures are known from the cave, as well as more than 70 non-figurative paintings representing geometric symbols (triangles, complex trapezoids, lines, “ladders”, etc.).

Following the classification of Leroi-Gourhan^6^, Bader tentatively referred the art to the late Solutrean (ca. 22—17 ka) to middle Magdalenian (ca. 17—12 ka) of the upper Palaeolithic^7^. Subsequently, an archaeological layer that contained fragments of ochre stencils as well as a fragment of painting on a piece of wall-rock was found in the cave, thus attesting the synchronicity of the layer and the artistic activity in the cave. Charcoal and bone material retrieved from this layer yielded radiocarbon dates of 16.3—19.6 ka (cal BP)^8–10^.

Although radiocarbon dates provide solid information on the timing of *some* artistic activity in Shulgan-Tash, the possibility that other paintings in the cave were made at different times remains. ^230^Th-U dating of calcite deposits underlying and overlying cave art has recently become the “gold standard” in this field^11–15^. We applied this method to constrain the minimum and the maximum ages of artistic activity in Shulgan-Tash. Another goal of our study was to reconstruct the environment in the cave during these artistic activities. In our unique approach we studied common speleothems (flowstone, stalagmites) and cryogenic cave calcite (CCC^16^) to constrain periods of time when liquid water was present in the cave (above-zero temperatures) and when deep cave interiors contained masses of ice (permafrost conditions) respectively.

## Results

### Dating the cave art

Paintings suitable for ^230^Th-U dating were found in the Domed Hall, Hall of Signs, and Hall of Chaos, all located in the lower level of the cave. Altogether, 58 individual ages at 22 locations were obtained (see Supplementary Information and ref.^17^). The “canvas” ages obtained from paintings in the Domed Hall and Hall of Signs, date to Marine Isotope Stages (MIS) 6 and 5 respectively: 138.6 ± 1.0 ka for painting #6-1 and 96.6 ± 0.5 ka for #12-1 (numeric identifications of paintings, preceded with number sign #, is according to^18^). Another MIS 5 age was obtained for pre-paint flowstone in the Hall of Chaos (#23-1; 95.7 ± 0.6 ka). Several samples of pre-paint flowstone in the Hall of Chaos indicate that calcite deposition terminated between 36.4 ± 0.1 and 44.1 ± 0.2 ka. Post-paint calcite deposition commenced in the early Holocene (e.g., 10.0 ± 0.5 ka ago for an unnamed red spot and 8.2 ± 0.5 ka ago for painting #15-1) in the Hall of Signs, and at the beginning of the Bølling-Allerød interstadial (14.3–14.5 ka ago; paintings #20-1, -2, -4, and -6) in the Hall of Chaos.

The ages of “canvas” and overgrowth calcite bracket large time windows when the paintings would have been created. The narrowest time window was identified in the Hall of Chaos (Table 1) where the youngest age of “canvas” flowstone is 36.4 ± 0.1 ka (#22-13) and the oldest age of overgrowing flowstone is 14.5 ± 0.06 ka (#20-1, -7). Similar ages were obtained for several other paintings in the Hall of Chaos. The minimum duration of the time window during which the paintings were made in this hall is therefore 21.9 ± 0.06 ka.

**Table 1:** Selected U and Th concentrations, isotopic activity ratios, and ages of samples from the Hall of Chaos, which provide the best constraint for the age of paintings. More details and additional results can be found in Supplementary Information. All measurements are reported with ±2σ absolute uncertainties.

Spl ID	Painting number and name*	^238^U		^232^Th		[^230^Th/^232^Th]_A_		δ^234^U^†^		[^230^Th/^238^U]_A_		^230^Th age (a BP)^‡^		^230^Th age (a BP)^‡§^	
		(ng/g)		(ng/g)		(measured)		(measured)		(measured)		(uncorrected)		(corrected)	
**Composition #20**															
YD01	20-4, Upper horse	1844.2	±4.8	65.7	±1.3	30	±1	246.0	±2.4	0.3720	±0.0015	38,196	±199	37,317	±610
YD02		1445.0	±3.4	29.2	±0.6	59	±1	287.1	±2.1	0.4183	±0.0015	42,202	±203	41,693	±374
ShT05-2	20-1, Lower horse	2262.7	±2.5	7.5	±0.2	166	±3	520.1	±1.7	0.1924	±0.0003	14,651	±32	14,523	±55
ShT05-1		1650.1	±1.6	52.2	±1.0	38	±1	275.6	±1.5	0.4156	±0.0008	42,344	±117	41,574	±512
ShT06-2	20-6, New anthropomorph	1823.7	±1.8	2.6	±0.05	377	±8	521.7	±1.7	0.1897	±0.0003	14,415	±31	14,322	±36
ShT06-1		1815.9	±2.5	89.8	±1.8	23	±1	239.3	±1.7	0.4038	±0.0008	42,407	±135	41,202	±817
**Slot**															
ShT09bis-2	22-7, Upper slingshot	4380.5	±6.9	2.1	±0.04	888	±18	181.6	±1.5	0.1485	±0.0004	14,600	±43	14,522	±44
ShT09bis-1		4127.8	±6.2	7.7	±0.2	562	±11	91.9	±1.5	0.3658	±0.0009	44,210	±148	44,095	±152
ShT10bis-5	22-12, Lower slingshot	5578.6	±9.3	1.3	±0.03	1621	±33	49.7	±1.3	0.1299	±0.0003	14,395	±43	14,322	±43
ShT10bis-4		7707.6	±15.3	0.5	±0.01	5940	±124	55.5	±1.5	0.1316	±0.0004	14,501	±52	14,433	±52
ShT10bis-3		6807.7	±13.5	5.1	±0.10	1054	±21	−49.9	±1.4	0.2776	±0.0008	37,801	±154	37,712	±154
ShT10bis-2		6918.3	±16.1	2.0	±0.04	2874	±59	−25.9	±1.6	0.2877	±0.001	38,239	±177	38,164	±177
ShT14b-9	22-13, Tower	3290.4	±4.3	3.5	±0.07	392	±8	214.6	±1.6	0.1473	±0.0003	14,042	±35	13,951	±39
ShT14b-8		3948.9	±4.5	0.2	±0.01	9601	±276	296.4	±1.5	0.1616	±0.0003	14,445	±34	14,378	±33
ShT14b-7		5577.8	±8.7	0.2	±0.01	10770	±385	319.6	±1.7	0.1653	±0.0004	14,516	±40	14,449	±40
ShT14b-6		5605.3	±8.7	0.4	±0.02	6322	±314	295.5	±1.5	0.1621	±0.0005	14,505	±47	14,437	±47
ShT14b-5		4338.3	±5.8	8.3	±0.2	469	±10	107.0	±1.4	0.3165	±0.0007	36,513	±109	36,397	±115
ShT14b-4		3783.5	±4.6	5.7	±0.1	648	±13	130.9	±1.4	0.3382	±0.0007	38,464	±110	38,361	±113
ShT14b-3		3190.3	±3.5	4.9	±0.1	660	±13	138.3	±1.4	0.3545	±0.0006	40,361	±105	40,257	±109

Subscript A denotes activity ratio.

*Painting number and names according to.^18^

^†^δ^234^U = ([^234^U/^238^U]_activity_ − 1)×1000.

^‡^[^230^Th/^238^U]_activity_ = 1 − e^−λ230T^ + (δ^234^U_measured_/1000)[λ_230_/(λ_230_ − λ_234_)](1 − e^−(λ230–λ234)T^), where T is age in years. λ_230_ = 9.1705 × 10^−6^ a^−1^ (ref.^27^), λ_234_ = 2.8221 × 10^−6^ a^−1^ (ref.^27^), λ_238_ = 1.551 × 10^−10^ a^−1^ (ref.^33^).

^§^Corrected for detrital Th contamination using an assumed initial [^230^Th/^232^Th]_A_ value of 0.8 ± 0.4 derived from the silicate bulk earth (ref.^32^). The degree of detrital ^230^Th contamination is indicated by the measured [^230^Th/^232^Th]_A._

BP stands for “Before Present” where the “Present” is defined as the year 1950 A.D.

### Dating speleothems

In order to better constrain past conditions inside the cave we studied two types of speleothems. Formation of “common” speleothems such as flowstone and stalagmites requires the presence of water films and dripping water in the cave. In contrast, cryogenic cave carbonates (CCC) form when cave passages are within the permafrost zone and cave air temperatures are slightly freezing, but overall, permafrost is degrading in response to climate warming^16^. Liquid water from the active layer and epikarst above the cave enters the karst, but forms ice upon reaching the cave chamber. During a long-term warming trend the cave temperature will stabilise at near-zero values, leading to the co-existence of water and ice. As the water in pools on the ice slowly freezes, it becomes progressively enriched in dissolved ions, eventually leading to the precipitation of CCC. ^230^Th-U dating of stalagmites and CCC therefore allows the identification of periods of both permafrost-free and permafrost conditions, respectively, in the cave and, by extension, in the study area. To this end, we collected 14 stalagmites from four caves located within 6 km (Shulgan-Tash, Victoria, Grioz, and Kulyurtamak caves^17^) and obtained 27 individual ages from them. Nine stalagmites started to grow in the mid-Holocene (from 7.7 ka onward). Five samples showed growth during MIS 5 and early MIS 4 (from 129 to 73 ka) and two samples during older periods (MIS 7, 8, 10 and 11). Importantly, of these sampled stalagmites, no deposition occurred between 73 and 7.7 ka (i.e. during MIS 2, 3 and most of MIS 4).

CCC were found in Shulgan-Tash and Victoria caves. We obtained 23 individual ages of 33–34, 41–42, 47, 53, 55 and 56 ka (see Supplementary Information).

## Discussion

**Figure 2:**
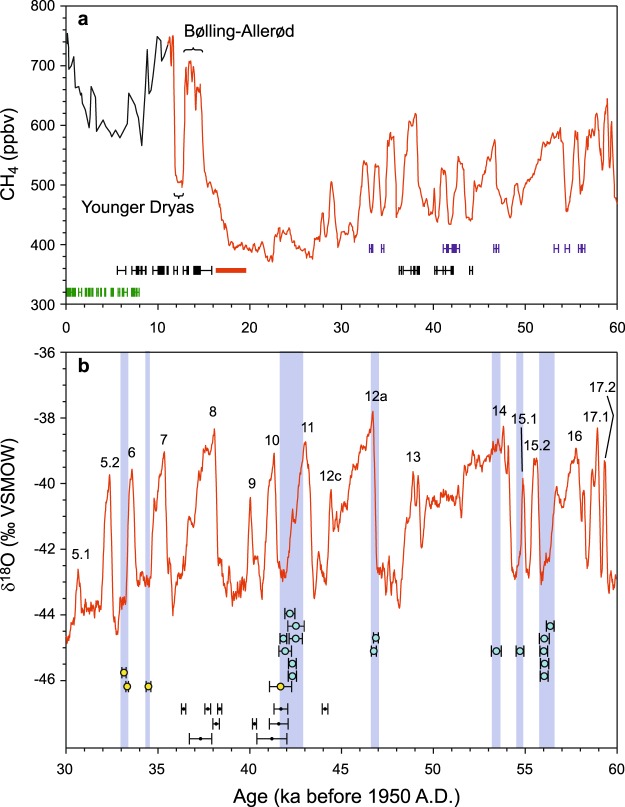
Speleothems as indicators of permafrost and permafrost-free conditions in the Southern Ural. (**a**) ^230^Th-U ages of stalagmites (green), pre- and post-art flowstone (black), cryogenic calcite (blue), and the range of calibrated ^14^C ages of charcoal from the cultural layer (red bar) superimposed on the long-term changes in methane concentrations as recorded in Greenland ice cores GISP2 (black line^35^ and NGRIP (red line^36^). Low concentrations of methane correspond to cold and dry climate, whereas high concentrations correspond to temperate humid conditions. (**b**) ^230^Th-U ages of cryogenic calcite from Shulgan-Tash (yellow dots) and Victoria caves (blue dots) as well as ages of pre-art flowstone (black dots) compared to the timing of MIS 3 Greenland interstadials (numbered) as expressed in the NGRIP δ^18^O record (red line^21^). Vertical blue bars mark times of growth of cryogenic cave calcite. ^230^Th-U and ^14^C ages are shown with corresponding 2σ uncertainties.

The parietal paintings in Shulgan-Tash cave made with red ochre are thought to have broadly similar ages^2,7,9,19^. Our ^230^Th-U dating demonstrates that the paintings on the walls of the Hall of Chaos were made between 36.4 ka to 14.5 ka. Four radiocarbon dates reported from the archaeological layer (16.5 to 19.6 ka cal BP) fall within the younger end of this 21.9 ka-long time window, constraining the time of at least some of the artistic activity in the cave to the last glacial termination (LGT), prior to the Bølling-Allerød interstadial (Fig. 2).

Our results indicate that the paintings were made on a dry canvas, i.e. during a depositional hiatus of the flowstone. This hiatus occurred synchronously with breaks in deposition of the stalagmites. Growth stops in such “common” speleothems may be caused by several factors, ranging in scale from local (e.g., changes in the drip site feeding the stalagmite) to intermediate (e.g., affecting the whole cave) to regional (affecting the region as a whole). Because our results were obtained from a number of speleothems collected in four different caves, we rule out local- and intermediate-scale growth controls and attribute the long hiatus during MIS 3 and 2 to cold and dry conditions prevailing in Southern Ural at that time.

The lack of stalagmite growth in Siberian caves during certain periods of the Pleistocene has been used as an indicator of past permafrost conditions^20^. Although this interpretation is warranted for areas that are presently located in the permafrost zone (such as Eastern Siberia), applying it to caves located outside of today’s permafrost zone, such as Southern Ural, requires additional justification. This is provided by CCC found in Shulgan-Tash and Victoria caves whose ages indicate that permafrost was temporarily present in the study area during MIS 3. Because CCC form under conditions of degrading permafrost, the spread in CCC ages suggests that the local permafrost was rather unstable during this time interval. At the depth of the studied caves (ca. 50–80 m below the surface), at least seven freeze-thaw cycles are recorded. Permafrost thawing episodes occurred in association with Greenland Interstadials^21^ (GI) 16, 15.1, 14, 12a, 11, 7 and 6 (Fig. 2). The relative timing with respect to the GIs varies: some CCC formed at or immediately after the peak warming (e.g., GI 15.1 and 12a); more commonly, however, CCC formed towards the end of interstadials and during the transition into stadials (GI 16, 11, 7 and 6). There might have been more cycles of permafrost freezing and thawing during MIS 3, but not all of them necessarily left traces in the form of CCC in the studied caves. Note that the very thin (<2 mm) pre-paint flowstone in the Hall of Chaos indicates short (1.5–2.5 ka-long) pulses of small-scale seepage flow (i.e. above-zero temperatures in the cave) associated with warmings during GI 8, 9, 10 and possibly GI 12 (Fig. 2).

Our results indicate that the but highly variable climate during MIS 3 was sufficiently cold to support permafrost in Southern Ural. The permafrost dynamically responded (thawed) to short-lived warmings of interstadials, as reflected by formation of CCC during these times. As MIS3 gave way to the significantly colder MIS 2, it is reasonable to assume that pervasive and continuous permafrost developed in the study area and persisted until the major warming at the onset of the Bølling-Allerød interstadial (Fig. 2). Apparently, the MIS 2 temperatures were so low that the short-term warmings associated with GI4 and 3 were not sufficient to cause formation of CCC or even ‘common’ speleothems in caves.

This means that during the time of artistic activity, i.e., between ca. 36.4 and 14.5 ka, Shulgan-Tash cave was an inhospitable place with below-zero air temperatures throughout the year and no dripping or running water. The paintings were made by Palaeolithic artists on cave walls that were dry^19^; our data indicate that the dryness was due to the freezing temperatures.

Our palaeoclimate and permafrost reconstruction is supported by data from other regional environmental archives. Studies of mammal assemblages suggest that environmental conditions in the Southern Ural during the Last Glacial Maximum (LGM, 28–20.5 ka) and LGT (20.5–15.5 ka) were characterised by severe cold climate and open periglacial landscapes, i.e. tundra and forest-steppe, with forests largely restricted to river valleys^22^. Osteological materials obtained from the 16.3–19.6 ka (cal BP) cultural layer in Shulgan-Tash cave comprised cold-adapted species characteristic of tundra, forest-tundra and steppe habitats: mammoth (*Mammuthus primigenius*), cave bear (*Ursus spelaeus*), mountain hare (*Lepus* sp.), polar fox (*Alopex lagopus*), marmot (*Marmota bobac*), pika (*Ochotona* sp.), and collar lemming (*Dicrostonyx* sp.)^23^. These findings are supported by palynological data from the same layer, featuring contorted pollen of Siberian spruce (*Picea obovata*), common pine (*Pinus sylvestris*), dwarf birch (*Betula humilis*), larch (*Larix sp*.), and juniper (*Juniperus sp*.). Grass and sage pollen are dominated by *Asteraceae*, while spores are represented by sphagnum moss (*Sphagnum sp*.) and Siberian lycopodium (*Lycopodium sibirica*)^19^. The palynological and osteological material from the cultural layer therefore indicates a cold environment with tundra vegetation and a fauna adapted to severe coldness.

At the time of the Last Permafrost Maximum (LPM; 25–17 ka), the southern boundary of equilibrium permafrost in this part of Eurasia was at about 48°N^24^. The study area was located in a zone of continuous permafrost, where the thickness of perennially frozen ground reached around 200 m deep, and the mean annual temperature was between −5 and −3 °C^24,25^. Subsequent to 17–15 ka, permafrost thawing began and its southern boundary retreated northward by ca. 400 km per century. Around 13 ka (Allerød), the boundary moved close to the Arctic Circle. During the Younger Dryas, the southern boundary of continuous permafrost advanced southward by 1700–1800 km, reaching the area of the southern Ural Mountains (51°N), followed by the final retreat of almost 2000 km during the mid-Holocene^26^.

### Final demise of permafrost in Shulgan-Tash

**Figure 3:**
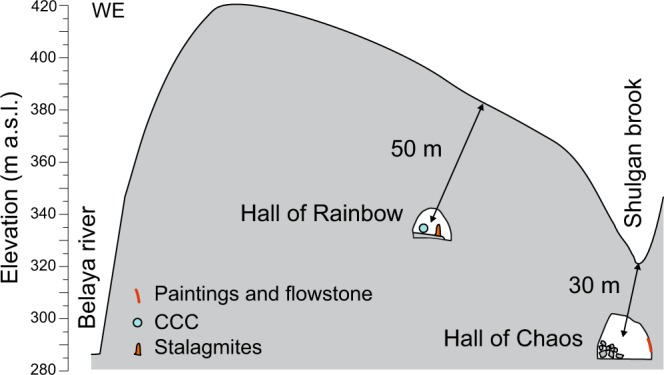
Schematic cross-section of the Saryk-Oskan massif. Locations of Shulgan-Tash cave chambers relative to the surface of the mountain are shown.

At the end of the LGM, flowstone and stalagmite growth commenced in different chambers of the cave at different times. At the lower level of the cave the earliest flowstone growth occurred at 14.5 ± 0.04 ka in the Hall of Chaos and around 10.0 ± 0.5 ka and 8.3 ± 0.4 ka in Halls of Signs and Domed (see Supplementary Information). Stalagmites in the upper galleries of the cave started growing even later, at 6.4 ± 0.3 ka. The period of no growth, therefore, appears to have been of different lengths in different parts of the cave. This can be explained by the 3D geometry of the cave in relation to the shape of the Saryk-Oskan massif, which controls the dynamics of permafrost thawing. Although the Hall of Chaos belongs to the lower level of the cave, it is located underneath the steep eastern slope of the massif incised by the gorge of the Shulgan brook (Fig. 3). This chamber, therefore, is closer to the surface (25–35 m) than the halls of the upper level of the cave (ca. 45–55 m of rock overburden). The Hall of Signs and Domed Hall are at intermediate positions. Since the degradation of permafrost during climate warming proceeds from the surface downward, it stands to reason that the Hall of Chaos, located closer to the surface, thawed first at ca. 14.5 ka thus allowing water to enter the cave and flowstone to form. Thawing of the thicker rock overburden above the halls and galleries of the upper cave level, where stalagmites were collected, took longer and the water reached this part of the cave only at ca. 6.4 ka. Warming associated with the Bølling-Allerød interstadial was sufficient to thaw the upper 25–35 m of permafrozen rock, allowing water to enter the Hall of Chaos (Fig. 3). Cave passages and halls located deeper thawed later, during the final demise of the permafrost in the Holocene. Similarly, wide-spread stalagmite growth in Victoria cave (at a depth of 75 to 90 m) only commenced at ca. 7.8 ka.

Similar logic explains an apparent inconsistency in Fig. 2, which suggests that at around 41–42 ka CCC and flowstone formed simultaneously. The thin layers of flowstone formed in halls on the lower level of the cave (located closer to the surface), whereas CCC formed on the upper level (underneath a thicker rock overburden). These data therefore demonstrate that cryogenic and “common” speleothem growth can occur simultaneously in different parts of the same cave.

Our study indicates that in the Southern Ural (latitude 53° N) permafrost conditions prevailed during most of MIS 3 and the entirety of MIS 2. Stalagmites and flowstones could not form during these times; their formation resumed on a very local scale only during the Bølling-Allerød interstadial. The onset of wide-spread speleothem formation occurred as late as the mid-Holocene.

Because of wide-spread permafrost, conditions in the Central and Northern Ural and in Siberia were likely even more restrictive with respect to speleothem formation during Late Palaeolithic times. Speleothem growth was possible only during the warmest interglacial periods (cf.^20^). One exception might be very shallow caves that could have been permafrost-free and allowed local growth of speleothems during short warm intervals, such as interstadials of MIS 3. The Upper Palaeolithic speleothem record therefore is expected to be fragmented (in both spatial and temporal senses) in northern Eurasia, and the potential of ^230^Th-U dating of speleothems for improving the chronology of Upper Palaeolithic archaeological cave sites in the Ural Mountains and Siberia therefore appears limited.

## Methods

### Sampling

Samples of flowstone associated with parietal art were collected either as powders *in situ*, using a hand-held milling device, or as 8 mm-diameter cores. Stalagmites were collected in those parts of the caves where their removal caused minimal visual impact. Sub-samples for ^230^Th-U dating (6 to 200 mg) were milled from the collected samples in the laboratory in a clean air laminar flow hood.

### ^230^Th-U dating

Dating was performed at three laboratories using Multi Collector Inductively Coupled Plasma Mass Spectrometric (MC-ICPMS) techniques. At University of Minnesota (USA), samples were spiked with a mixed ^229^Th-^233^U-^236^U tracer prior to chemical separation of U and Th using an Fe co-precipitation procedure followed by chemical purification on anion exchange columns^27,28^. Procedural chemistry blanks were typically less than 100 at for ^230^Th and less than 1 fg for ^234^U. U and Th isotopic ratios and concentrations were determined using the latest protocols on a ThermoFisher Neptune MC-ICPMS^28^ and the half-lives from^29^. At Max Planck Institute for Chemistry (Germany), chemical separation of U and Th isotopes was performed as described in^30^. U and Th isotopes were analysed using a Nu Plasma MC-ICPMS. Analytical details are described in^31^. Details about the calibration of the mixed U-Th spike are given in^32^. All activity ratios were calculated using the half-lives from^33^, and all ages are reported at the 2σ-level. At Xi’an Jiaotong University (China), analyses followed chemistry procedures described in^27^ to separate uranium and thorium. U and Th isotopes were analysed individually by using a ThermoFisher Neptune Plus MC-ICPMS as described in^28,29^. Age corrections assume an initial ^230^Th/^232^Th ratio of 4.4 ± 2.2×10^−6^ of bulk Earth^34^.

### Data availability

A document detailing the sampling procedure associated with this project is deposited in the Mendeley Data open research data repository (Dublyansky and Lyakhnitsky, 2018). All other data are included in the Supplementary Material or available upon request to the corresponding authors.

## Electronic supplementary material

Dataset 1
